# A Critical Analysis of Representations of Inequalities in Childhood Obesity in Australian Health Policy Documents

**DOI:** 10.34172/ijhpm.2021.82

**Published:** 2021-08-07

**Authors:** Alexandra Chung, Christina Zorbas, Anna Peeters, Kathryn Backholer, Jennifer Browne

**Affiliations:** ^1^School of Public Health and Preventive Medicine, Monash University, Melbourne, VIC, Australia.; ^2^Global Obesity Centre (GLOBE), Institute for Health Transformation, Deakin University, Geelong, VIC, Australia.

**Keywords:** Child Obesity, Obesity Prevention, Health Inequities, Health Equity, Health Policy, Australia

## Abstract

**Background:** In Australia, childhood obesity follows a socioeconomic gradient whereby children with lower socioeconomic position are disproportionately burdened. To reduce these inequalities in childhood obesity requires a multi-component policy-driven response. Action to address health issues is underpinned by the ways in which they are represented as ‘problems’ in public policy. This study critically examines representations of inequalities in childhood obesity within Australian health policy documents published between 2000-2019.

**Methods:** Australia’s federal, state and territory government health department websites were searched for health policy documents including healthy weight, obesity, healthy eating, food and nutrition strategies; child and youth health strategies; and broader health and wellbeing, prevention and health promotion policies that proposed objectives or strategies for childhood obesity prevention. Thematic analysis of eligible documents was guided by a theoretical framework informed by problematization theory, ecological systems theory, and theoretical principles for equity in health policy.

**Results:** Eighteen policy documents were eligible for inclusion. The dominant representation of inequalities in childhood obesity was one of individual responsibility. The social determinants of inequalities in childhood obesity were acknowledged, yet policy actions predominantly focused on individual determinants. Equity was positioned as a principle of policy documents but was seldom mentioned in policy actions.

**Conclusion:** Current representations of inequalities in childhood obesity in Australian health policy documents do not adequately address the underlying causes of health inequities. In order to reduce inequalities in childhood obesity future policies will need greater focus on health equity and the social determinants of health (SDoH).

## Background

**Key Messages**

** Implications for policy makers**

- The dominant representation of inequalities in childhood obesity in Australian health policy documents was one of individual responsibility with policy actions predominantly focused on individual determinants of health behaviour.
- Actions to address underlying drivers of obesity are necessary to shape health-promoting environments which support, instead of undermine, healthy behaviours, however this was not a focus of actions proposed in the Australian health policy documents analysed in this study.
- Current representations of inequalities in childhood obesity in Australian health policy documents do not adequately address the underlying causes of health inequities. In order to reduce inequalities in childhood obesity future policies will need greater focus on health equity and the social determinants of health (SDoH).

** Implications for the public**

This critical analysis of a sample of 18 Australian national, state and territory health policy documents explored how governments consider socioeconomic inequalities in childhood obesity in public health policy documents. Findings indicated childhood obesity is predominantly represented as an issue of individual responsibility in Australian national, state and territory public health policy documents. Actions proposed in policy documents focused primarily on improving knowledge and skills and changing children’s dietary behaviour. These actions alone are unlikely to address the underlying causes of childhood obesity and may widen inequalities in childhood obesity. In order to better promote fair opportunities for health, policies need a greater focus on health equity, necessitating action on the social determinants of health (SDoH) within and beyond the health system.

Children with obesity have increased risk of adverse physical and psychological health, with excess weight gain and associated health consequences likely to persist into adolescence and adulthood.^1-4^ In Australia and other high-income countries, childhood obesity is socioeconomically patterned whereby children who live in socioeconomically disadvantaged neighbourhoods or whose parents have low income or education are more likely to experience obesity compared to children with a relatively higher socioeconomic position.^5,6^ In a number of countries including Australia, socioeconomic inequalities in childhood obesity are widening.^7,8^

 The determinants of childhood obesity are complex, acting across multiple contexts and in multiple settings.^9^ Childhood overweight and obesity is influenced by individual factors including diet and physical activity, knowledge and skills, individual preferences and parenting practices.^10,11^ These individual factors are in turn shaped by the social determinants of health (SDoH), the daily living conditions in which we are born, grow, work, live, play and age.^11,12^ They include early life experiences, education and employment opportunities, housing, and food environments.^12^ The SDoH also include the economic, social, political and cultural contexts that shape these daily living conditions.^13^ The SDoH are not experienced equally with some populations having less access to the social and economic resources and conditions necessary for good health.^14^ These differences in the SDoH give rise to unjust inequalities in health.^13^ Health *equity* therefore can be described as the notion that everyone should have a fair opportunity to attain their full health potential.^15^

 Implementation of comprehensive and multi-faceted government policies across all levels of influence is necessary to create and support healthy and equitable food and physical activity environments.^14,16,17^ Evidence shows that in order to be both effective and equitable, actions must address the structural barriers to good health and a healthy weight.^18^ Interventions that target structural barriers to health aim to change the circumstances in which individuals’ decisions are made, in order to better support healthy choices.^18^ Unlike behavioural interventions, these do not require individual agency and are typically enacted through regulatory changes.^18^

 Central to equity-oriented health policy is its attention to ensuring less privileged groups have the same opportunities to attain the same level of health as those who are better off.^19^ Carefully designed policies that address the underlying drivers of obesity and create health promoting environments combined with targeted actions that are proportionate to need can bring about fairer opportunities for good health across societies.^18^

 It is widely recognised that there is a role for government policy in obesity prevention.^20-22^ Government policies reflect policy-makers’ ideologies and values and are shaped by political, institutional and interest groups.^23^ They carry implicit or explicit problem representations that both influence, and are influenced by, media and public discourse and opinion, and can further shape the political agenda.^24^ The way issues are represented as problems within policy documents can be considered alongside the way in which policy actors frame issues. Problem representation analysis begins with proposed solutions and examines the implicit problem(s) within those solutions.^25^ Framing, on the other hand examines how language is used by stakeholders to construct reality, through shaping perceptions of an issue it is causes and solutions.^26^ Problem representations and issue framing within policies can direct attention to, or shift the focus from, particular aspects of a problem, which in turn influences the actions that are available to address the problem.^25^ Analysis of how policy problems are conceptualized provides the opportunity to rethink current, or inform future, policy, research and advocacy efforts.^27^

 This study focuses on health policy documents as an indicator of a government’s institutional commitment to act on inequalities in childhood obesity.^28^ To date there have been no previous analyses of representations of inequalities in childhood obesity in health policy. The aim of the study, therefore, is to understand how inequalities in childhood overweight and obesity are represented as a problem in Australian health policy documents.

## Methods

###  Study Setting

 Australia has a federated governance system, comprising a federal (national) government and eight state/territory governments. Under this structure, both federal and state/territory governments are responsible for health and health promotion policy. As a consequence, obesity prevention is addressed in different ways across jurisdictions with some governments implementing specific healthy weight or obesity strategies, while others address childhood obesity within healthy eating or food and nutrition strategies; child and youth health strategies; or broader health and wellbeing, prevention or health promotion policies.

###  Study Design

 Qualitative policy analysis was undertaken to critically analyse public health and obesity prevention policy documents published by Australia’s national, state and territory governments. We adopted an interpretive approach to policy analysis which is particularly useful for interrogating the framing, representation and social construction of policy problems.^27^ We used a theory-informed approach to examine how (1) childhood obesity and (2) inequalities in childhood obesity have been represented as problems in policy actions to prevent childhood obesity proposed during the period 2000-2019.

###  Theoretical Perspective

 Multiple theories and principles relevant to health policy analysis were used to inform specific components of our methodological approach. These included problematization theory, ecological systems theory, and theoretical principles for advancing equity in health policy. We combined these theoretical perspectives to develop a comprehensive analytic framework (Table 1).

**Table 1:** Coding and Theoretical Analysis Framework

**Theoretical Perspective **	**Description**	**Guiding Questions for Data Coding and Analysis**	**Codes**
Problematization theory, applied as WPR^29^	The WPR approach draws on problematization theory and comprises critical analysis questions to interrogate policy recommendations or actions, ultimately identifying how problems are implicitly or explicitly represented within these.	1. What’s the problem represented to be in a specific policy or policies? 2. How has this representation of the “problem” come about? 3. What is left unproblematic in this problem representation? Where are the silences? 4. What effects are produced by this representation of the “problem”?	Health burden Disease prevention Economic impacts Unhealthy behaviours Lifestyles Vulnerable populations Individual responsibility
Ecological systems theory^40^	According to ecological systems theory, health is influenced by multiple factors operating across several levels. Dahlgren and Whitehead align four levels of policy action to the corresponding determinants of health.	1. How are the determinants of childhood obesity represented across (*i*) macro (*ii*) settings (*iii*) community (*iv*) individual levels? 2. How do proposed policy actions align across (*i*) macro, (*ii*) settings, (*iii*) community and (iv) individual levels?	Individual choice Behaviour not meeting guidelines Unhealthy food marketing Obesogenic environments SDoH Unhealthy norms Individual responsibility Education and information Increase fruit and veg intake Social marketing School programs Sports and recreation policies Government responsibility Shared responsibility Partnerships
Key concepts and principles for promoting equity in health policy^34,35^	Evidence-based features of a policy response to promote equity or reduce health inequalities.	1. How have equity objectives and targets been described in the policy? 2. To what extent are actions to prevent childhood obesity targeting the SDoH inequalities? 3. How does the policy report on or plan for measurement of inequalities and outcomes for different socioeconomic groups?	Equity in principle Equity objectives Equity targets Whole of populations Social determinants Targeted interventions Priority populations Research and monitoring

Abbreviations: SDoH, social determinants of health; WPR, What’s the Problem Represented to be.

 Based on problematization theory, Bacchi’s ‘What’s the Problem Represented to be’ (WPR) framework was used as the overarching approach to guide the analysis.^25,29^ WPR proposes that analysis of problem representations must begin with the policy actions – which implicitly or explicitly represent dominant ideas, values and priorities, and silences across these domains. For example, proposing nutrition education to address childhood obesity indicates that obesity arises from knowledge and skill deficits.^25^ Yet, this silences the remaining socioecological causes of obesity, risking the deflection of political action across these levels.

 The WPR approach is underpinned by Foucault’s theory of problematization, which suggests that policies contain implicit representations of problems within the strategies they propose.^29^ Bacchi observes that “we are governed through problematizations rather than policies.”^29^ The WPR approach thus provides an analytic strategy that can support critical interrogation of policy documents to reveal how issues become defined as “problems” and the political values and assumptions underlying these problem representations.^25^

 Previous studies have demonstrated that WPR is a useful approach for interrogating how public health nutrition and childhood obesity are problematized in policy documents and media stories.^30-33^ For example, analysis of Australia’s now expired national public health nutrition agenda, Eat Well Australia, revealed public health nutrition was represented as an individual problem, and as a problem arising from social, political and economic circumstances.^30^ Analysis of media representations of childhood obesity in Australia revealed media coverage favoured the representation of childhood obesity as a problem of individuals, in direct contrast to the social representation observed in the academic literature.^33^

**Figure 1:**
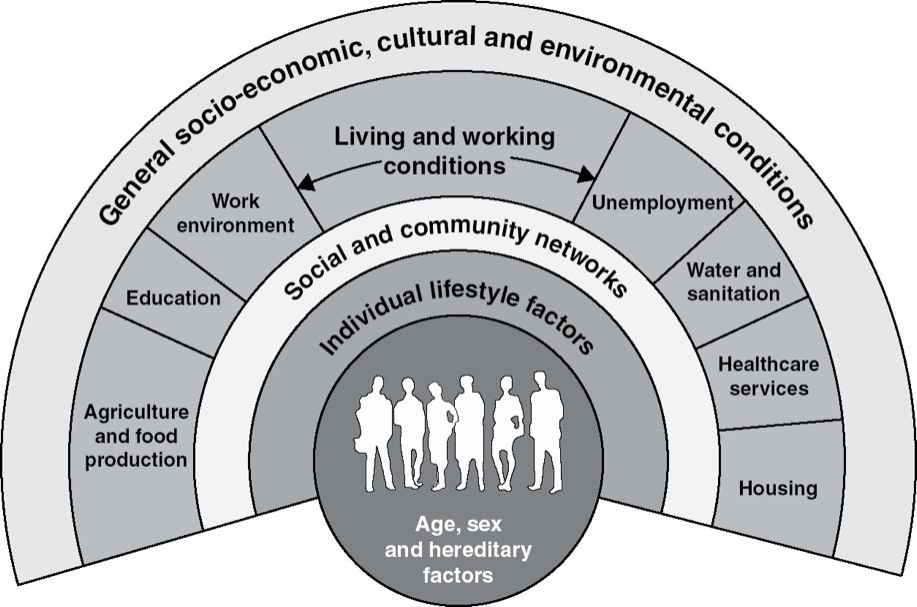
Determinants of Health Model. Source: Dahlgren and Whitehead (1991).^19^

 To examine the representations of childhood obesity and consider the equity impacts of proposed actions, we were guided by ecological systems theory in developing our analytic framework. In particular, we drew upon Dahlgren and Whitehead’s application of ecological systems theory.^19^ In their model, the outermost layer represents the socioeconomic, cultural and environmental determinants of health (level 1); moving inward, the next layer represents the settings in which people live, work, and learn (level 2); the next layer represents family, social and community networks (level 3); and the innermost layer represents individual behaviours and biological factors (level 4) (Figure 1).^19^ These four levels of influence translate into corresponding levels of policy action. Level one policy actions bring about structural change, such as taxation policies. Level two policy actions improve daily life conditions, for example through employment and protection policies, or food and nutrition policies that improve access to and affordability of healthy foods. Level three policy actions build community capacity though improving local skills, leadership and infrastructure to better enable implementation of tailored actions that improve health within the community. Finally, level four policy actions influence individual behaviour, such as nutrition labelling or providing health education.^19^

 To further interrogate the extent to which policy documents address equity, the analytic framework drew upon Whitehead and Dahlgren’s Ten principles for policy action^34^ and Whitehead, Dahlgren and Gilson’s key action areas for robust policy response to health inequalities.^35^ Guiding questions for analysis were selected based on relevance to this particular critical analysis of policy documents from a single country. These documents outline key evidence-based policy features that are considered essential to promote equity in health policy^14,36^ and have previously been applied to understand inequities in children’s health including obesity,^37,38^ and in frameworks to examine health equity in child health policies.^39^

###  Reflexivity Statement

 All researchers involved in this study are white, female and tertiary educated, with experience identifying and analysing policies to equitably reduce non-communicable diseases. Our professional backgrounds include the fields of nutrition and dietetics, chronic disease prevention, epidemiology, health equity, health policy, and Aboriginal and Torres Strait Islander health. In accordance with existing theories and evidence, we are of the view that health and health inequities are influenced by the social determinants that shape the conditions in which individual behaviours occur.

###  Document Selection

 For the purpose of this study we were interested in government health policy documents in order to analyse policy representations of inequalities in childhood overweight and obesity. Each of Australia’s nine national, state and territory government health department websites were searched by one researcher (AC) between October 2019 and December 2019 for potentially relevant health policy documents. These included (1) national or state or territory healthy eating/ obesity prevention policy documents, and** (**2) national or state or territory public health policy documents (including health and wellbeing, prevention, health promotion, and child and youth health policy documents) that proposed objectives or strategies for childhood obesity prevention, as an indication of governments’ intentions. This range of document types was sought because of the observed inconsistencies in obesity prevention policy documents produced by national, state and territory governments. Keyword searches (for child, childhood, obesity, weight) were conducted to identify if a document was relevant to the study.

###  Inclusion and Exclusion Criteria

 To be eligible for inclusion, documents had to be (*i*) published by a government health department, (*ii*) a policy, strategy, strategic framework, plan, strategic plan, or action plan, (*iii*) published between 2000 and 2019 (to capture current or most recent childhood obesity prevention policy proposals), and (*iv*) explicitly outline objectives or strategies for preventing childhood obesity. Only health department policies were eligible because health departments are likely to take the lead/coordinating role for policies aiming to reduce childhood obesity. Documents were excluded if they did not outline actions specifically pertaining to the prevention of child obesity, or for which a more recent version of the same document was available.

###  Data Extraction

 The following details were entered into a data extraction matrix developed for this study in Microsoft Excel: the title of the policy; level of government (state/territory or national); the type of the policy (eg, public health plan, healthy eating strategy); the timeframe of the policy; the overall goal of the policy.

###  Data Analysis 

 The representations of childhood obesity and inequalities in childhood obesity in Australian health policy documents were examined through a theory-informed analysis. Policy documents were read in full and all sections relating to the prevention of childhood overweight and obesity were inductively coded, guided by the questions outlined in the analytical framework (Table 1). Guiding principles and overarching statements in each policy document were inductively coded in the same manner. Two authors (AC and CZ) independently coded a subset of three policy documents, before comparing, labelling and defining codes and developing a coding framework that was used to code the remaining documents by a single author (AC) (Table 1).

 Codes were subsequently aggregated to generate higher-order themes illustrating the representations of inequalities in childhood obesity.^41^ To do this, text-based thematic maps were constructed to iteratively explore relationships between codes and the theory, using the guiding questions from the analytic framework.

 Final themes were verified in three ways; (1) mapping the codes against the analytic framework; (2) revisiting the policy documents throughout the analytical process; and (3) through discussion with all members of the research team.^42^

## Results

**Figure 2:**
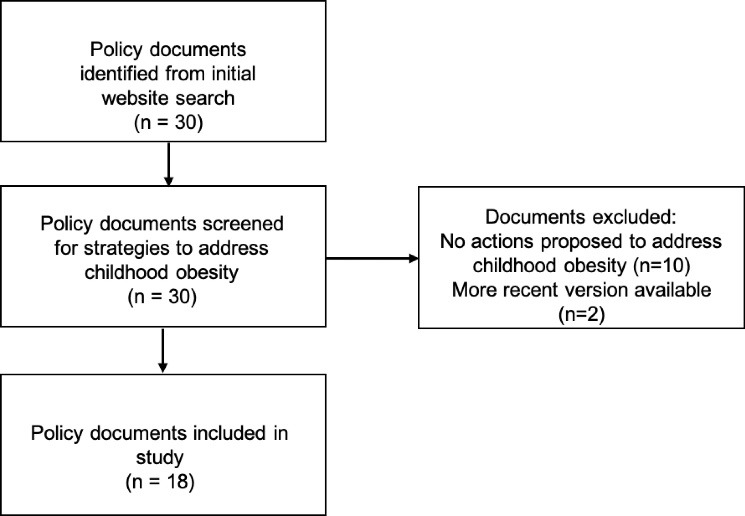
Article Selection Flow Diagram.

 A total of 30 documents were retrieved, of which 18 were eligible for inclusion in the analysis. Excluded documents either did not outline specific actions to address childhood obesity prevention, or a more recent version was identified. A flow chart illustrating the document selection process is shown in Figure 2. Nine policy documents focussed on healthy weight or obesity (n = 4), healthy eating (n = 4), food and nutrition (n = 1) were included from national (n = 2) and state and territory (n = 7) governments. A further nine public health policy documents outlining strategies for health and wellbeing (n = 4), prevention (n = 1), health promotion (n = 1), child and youth health (n = 3) were included from national (n=2) and state and territory governments (n=7). Details including titles and jurisdictions of included documents is provided in Table 2.

**Table 2:** Policy Documents Included in the Analysis and Political Party in Government at Time of Publication

	**Public Health Strategies**	**Obesity/Healthy Eating Strategies**
**Australia**	National Preventative Health Strategy: Australia the healthiest country by 2020 *Published 2009, Australian Labor Party*	
	Healthy Safe and Thriving: National Strategic Framework for Child and Youth Health 2015 *Published 2015, Liberal-Nationals Coalition*	
		Eat Well Australia: An Agenda for Action for Public Health Nutrition 2000-2010 *Published 2001, Australian Labor Party*
		Healthy Weight 2008: The National Action Agenda for Children and Young People and their Families *Published 2003, Liberal-Nationals Coalition*
**Victoria**	Victorian Public Health and Wellbeing Plan 2019-2023 *Published 2019, Labor Party*	
**NSW**	Healthy Safe and Well Strategic Health Plan for Children Young People and Families 2014-2024 *Published 2014, Liberal Party*	NSW Healthy Eating and Active Living Strategy: Preventing overweight and obesity in New South Wales 2013-2018 *Published 2013, Liberal Party*
		NSW Premier’s Priority: Reduce Overweight and Obesity Rates of Children by 5% over 10 years *Published 2016, Liberal Party*
**ACT**		ACT Towards Zero Growth Healthy Weight Action Plan *Published 2013, Labor Party*
**QLD**	Queensland Health and Wellbeing Strategic Framework 2017-2026 *Published 2018, Labor Party*	Queensland Health: Healthy Weight Strategy 2017-2020 *Published 2017, Labor Party*
**NT**	Northern Territory Child and Adolescent Health and Wellbeing Strategic Plan 2018-2028 *Published 2018, Labor Party*	Northern Territory Health Nutrition and Physical Activity Strategy 2015-2020 *Published 2015, Country Liberal Party*
**WA**	Western Australian Health Promotion Strategic Framework 2017-2021 *Published 2017, Labor Party*	
	Western Australian State Public Health Plan 2019-2024 *Published 2017, Labor Party*	
**SA**		The Eat Well Be Active Strategy for South Australia 2011-2016 *Published 2011, Labor Party*
**Tasmania**	Healthy Tasmania Five Year Strategic Plan 2016 *Published 2016, Liberal Party*	Tasmanian Food and Nutrition Policy 2004 *Published 2004, Labor Party*

Abbreviations: NSW, New South Wales; ACT, Australian Capital Territory; QLD, Queensland; NT, Northern Territory; WA, Western Australia; SA, South Australia. Liberal party core values include individual freedom and minimal state involvement; Labor party core values include social security and a balance between market economy and state intervention.^44^

 A total of six themes were identified, illustrating representations of inequalities in childhood overweight and obesity in Australian health policy documents (Table 3). Policy documents dedicated to healthy eating and obesity prevention proposed actions to prevention childhood obesity in more detail compared to general public health policy documents (including health and wellbeing, prevention, health promotion, and child and youth health policy documents). An overview of each of the themes is provided below, using excerpts from policy documents to illustrate how data contributed to each theme.

**Table 3:** Codes and Final Themes

**Codes**	**Themes**
Health burden Disease prevention Economic impacts	Childhood obesity is a burden
Unhealthy behaviours Lifestyles Individual choice Behaviour not meeting guidelines Vulnerable populations Individual responsibility Unhealthy norms Education and information Increase fruit and veg intake Social marketing	Childhood overweight and obesity is a problem of individual responsibility
Unhealthy food marketing Obesogenic environments SDoH School programs Sports and recreation policies	Social determinants contribute to childhood overweight and obesity
Individual responsibility Government responsibility Shared responsibility Partnerships	Shifting responsibility for childhood obesity prevention
Equity in principle Equity objectives Equity targets Research and monitoring	Equity in principle but not in practice
Whole of populations Social determinants Targeted interventions Priority populations	Priority populations for obesity prevention

Abbreviation: SDoH, social determinants of health.

###  1. Childhood Obesity Is a Burden

 Throughout policy documents childhood obesity was consistently represented as burdensome to the health system, the economy and to society. The burden of high rates of childhood obesity underscored the focus on childhood overweight and obesity. Objectives to reduce the burden of childhood overweight and obesity were made, and the health, economic and social burden of overweight and obesity was cited as a leading reason to act;

*“Based on the available evidence, it is very likely that the impact of overweight and obesity on quality of life, primary healthcare and the ACT economy are growing. The ACT community cannot afford inaction”* (ACT Towards Zero Growth Healthy Weight Action Plan, p. 5).

 Obesity was problematized as placing a higher burden on the health of particular population groups including people living in socioeconomically disadvantaged areas, regional and remote areas, and Aboriginal and Torres Strait Islander people. Policy documents included broad objectives to reduce childhood overweight and obesity (eg, Healthy Weight 2008; New South Wales [NSW] Premier’s Priority; Australian Capital Territory [ACT] Towards Zero Growth Healthy Weight Action Plan) and a small number of policies set overall targets for obesity prevention or reductio (eg, Queensland [QLD]Health Weight Strategy), however explicit targets for reducing childhood obesity among specific population subgroups were not identified. Furthermore, no targets were set for reducing inequalities in childhood obesity.

###  2. Equity in Principle but Not in Practice

 Equity was acknowledged in the majority of policy documents, commonly positioned as a guiding principle for the objectives and actions within. A number of equity-related concepts were theoretically discussed including health disparities, health inequalities, and social and economic disadvantage. Within these discussions, equity was more commonly represented as an issue of health differences than as an issue of fairness. Only a minority of documents including the Northern Territory Health Nutrition and Physical Activity Strategy and Victorian Public Health and Wellbeing Plan spoke to the issue of fairness;

*“This strategy acknowledges the need to address the multiple underlying social, economic and cultural determinants of health and aims to reduce health disparities seen in the NT, by focussing on those who experience the greatest disadvantage and are most at risk*” (Northern Territory Health Nutrition and Physical Activity Strategy 2015-2020, p. 7).

*“A fairer society is fundamental to improving the health of the whole population, yet we know that good health and wellbeing is not equally distributed across the population. Those who live with greater social and economic disadvantages are more likely to experience health inequalities”* (Victorian Public Health and Wellbeing Plan 2019-2023, p. 15).

 Most policy documents failed to report on the magnitude of inequalities in childhood overweight and obesity, with childhood obesity prevalence, trends and risk factor data predominantly reported in aggregate. Where childhood obesity data were reported by subgroup, this included reporting by age and/or sex, but not by indicators of socioeconomic position, silencing the health disparities within the population. One exception was seen in the NT Health Nutrition and Physical Activity Strategy which reported trends in overweight and obesity and health behaviours according to urban non-Aboriginal, urban Aboriginal and remote Aboriginal status. However, targets and actions to address the demonstrated differences were not proposed.

 Very few policy documents explicitly proposed action to reduce inequalities in childhood overweight or obesity. This indicates that such inequalities were insufficiently problematized as an issue in the health policy documents examined in this study. Although specific actions were seldom articulated, broad intentions to reduce inequalities were stated such as in the National Strategic Framework for Child and Youth Health;

*“Work collaboratively with other agencies and community health bodies to reduce disadvantage as a result of social determinants of health” *(Healthy Safe and Thriving: National Strategic Framework for Child and Youth Health 2015, p. 30).

 Equity objectives and targets were not articulated and consideration of equity impacts was lacking in actions proposed in policy documents. This leaves the impact of policies on different population groups largely unknown. A very small number of policies proposed monitoring of obesity, diet and physical activity indicators by sociodemographic characteristics as seen in the following example;

 “*Assess changes in prevalence of overweight and obesity, physical activity, healthy and unhealthy food consumption by sociodemographic groups (sex, age, socioeconomic status, remoteness and hospital *&* health services) for adults and children*” (Queensland Health: Healthy Weight Strategy 2017-2020, p. 8).

###  3. Childhood Overweight and Obesity Is a Problem of Individual Responsibility

 The representation of childhood overweight and obesity as a problem of individual responsibility was the dominant theme throughout the policy documents analysed in this study. This representation was evident in all policy documents, particularly in the causes of childhood obesity and through the solutions proposed within policy documents.

 The focus on the individual was evident through an emphasis on policy actions targeting individual behaviour. The dominant message throughout all policy documents was that individuals and their inadequate dietary and activity behaviours are primarily responsible for the high prevalence of childhood overweight and obesity in Australia. A number of policy documents (eg, Healthy Weight 2008; QLD Healthy Weight Strategy; NSW Healthy Eating Active Living Strategy; NSW Premier’s Priority; Victorian Public Health and Wellbeing Plan; Tasmanian Food and Nutrition Policy; Eat Well Be Active SA; WA Health Promotion Strategic Framework; NT Health Nutrition and Physical Activity Strategy) reported discordance between national guidelines and children’s behaviour such as consumption of unhealthy foods and sugar-sweetened beverages, and the proportion of children engaging in physical activity and sedentary behaviour, as illustrated by the following example;

 “*Non-observance with the Australian Dietary Guidelines was greatest in relation to vegetables, saturated fat and sugar for (children of) all age groups, as well as fruit and dairy intake for those 9 years and over*” (Northern Territory Health Nutrition and Physical Activity Strategy 2015-2020, p. 29).

 Policy documents (eg, QLD Healthy Weight Strategy; ACT Towards Zero Growth; Eat Well Be Active SA; WA Health Promotion Strategic Framework) also problematized “lifestyle choices” of individuals and families as responsible for childhood overweight and obesity;

 “*Compared to major cities, adult obesity rates are 22% higher in outer regional and 36% higher in remote and very remote areas. While disparities are not as evident among children, it is likely that family lifestyle choices over the longer term will put children at risk of weight gain*” (QLD Healthy Weight Strategy 2017-2020, p. 2).

 Maintaining a healthy lifestyle or preventing lifestyle-related ill health was commonly recommended as a strategy to prevent childhood obesity. All policy documents proposed objectives or actions at the individual level such as dietary and physical activity behaviour change, reinforcing the representation of childhood obesity as an individual problem. Actions such as information provision, education, and raising awareness were recommended in all policy documents. These actions problematize childhood obesity as an issue caused by individual choices, particularly parents’ choices, and as arising from a lack of knowledge or awareness;

*“Develop and disseminate information resources for parents at different stages of their child’s development – starting with new parents – on healthy eating, active living and healthy weight for themselves as well as their child”* (Healthy Weight 2008: The National Action Agenda for Children and Young People and their Families, p. 11).

###  4. Social Determinants Contribute to Childhood Overweight and Obesity 

 An alternative to the individual responsibility representation, is one that considers the underlying social determinants of childhood obesity. Compared to individual-level actions, policy documents were less likely to problematize childhood overweight and obesity as a problem to be addressed through structural interventions targeting the SDoH. It was common, however, for policy documents (eg, National Preventive Health Strategy; NSW Healthy Eating Active Living Strategy; Victorian Public Health and Wellbeing Plan; Eat Well Be Active SA; WA Health Promotion Strategic Framework) to rhetorically acknowledge the SDoH as drivers of childhood obesity;

 “*There is widespread consensus that the rise in overweight and obesity is mostly a result of social, environmental and technological changes over the last few decades. These changes have led to environments which encourage excess energy intake and reduced energy expenditure*” (New South Wales Healthy Eating and Active Living Strategy 2013-2018, p. 27).

*“We have recognised for some time that there is a broad range of social determinants that influence people’s wellbeing. How much you earn, the local environment, whether you have a job or are able to access the services you require will all have an impact on your diet, levels of physical activity, health, educational attainment, ability to secure and sustain housing, and risk of involvement with the criminal justice system” *(The Eat Well Be Active Strategy for South Australia 2011-2016, p. 5).

 Policy documents frequently problematised childhood overweight and obesity as a settings-based issue through a focus on actions within school or sport and recreation settings (eg, NSW Healthy Safe and Well; NSW Premier’s Priority; ACT Towards Zero Growth Healthy Weight Action Plan; Victorian Public Health and Wellbeing Plan; Eat Well Be Active Strategy for SA; NT Child and Adolescent Health and Wellbeing Strategic Plan; NT Health Nutrition and Physical Activity Strategy). Although this suggests a shift away from individual responsibility, settings-based actions frequently included those which rely upon individual agency such as nutrition education in school curricula, reinforcing the problematization of individual behaviour. On the other hand, proposed actions at the settings level also included actions to change the environment within those settings such implementing healthy menus within sport and recreation centres and food and nutrition policies in schools;

*“Develop and implement an ACT Government school food and drink policy with supporting guidelines that will mandate the implementation of the National Health School Canteen Guidelines in all ACT schools” *(ACT Towards Zero Growth Healthy Weight Action Plan, p.16).

 Proposed structural interventions included increasing availability and access to healthy food in communities, increasing the availability of free drinking water, and industry-led reductions in serving sizes and product reformulation. However, these actions were often loosely described leaving unclear the roles and responsibilities for implementation;

*“Encourage the food service industry to limit size of servings and reduce energy content of less healthy meals and snacks, and support the food industry to develop less energy dense products”* (Healthy Weight 2008: The National Action Agenda for Children and Young People and their Families, p. 14).

 Children’s exposure to unhealthy food marketing was commonly problematized as an issue, across a majority of policy documents. The National Preventive Health Strategy proposed detailed actions to address unhealthy food marketing;

*“Reduce exposure of children and others to marketing, advertising, promotion and sponsorship of energy-dense nutrient-poor foods and beverages.” Strategies include monitor and evaluate Industry self-regulation, identify shortfalls, introduce co-regulation, monitor, introduce legislation if self- and co-regulation are not demonstrated to be effective”* (National Preventive Health Strategy: Australia the healthiest country by 2020, p. 16).

###  5. Priority Populations for Obesity Prevention

 Priority populations were identified in all policy documents according to sociodemographic characteristics and included Aboriginal and Torres Strait Islander peoples, culturally and linguistically diverse communities, socioeconomically disadvantaged communities and mothers and young children. The identification of priority groups was frequently seen in policy rhetoric and less commonly problematised through the proposal of actions targeted towards particular groups.

 Policy documents commonly referred to population groups as “disadvantaged” (eg, QLD Health and Wellbeing Strategic Framework; ACT Healthy Weight Action Plan; Eat Well Be Active SA; NT Health Nutrition and Physical Activity Strategy) or “vulnerable” (eg, Eat Well Be Active SA; Tasmanian Food and Nutrition Policy; WA Health Promotion Strategic Framework) to poor health. Documents inconsistently elaborated which population groups experience disadvantage or vulnerability and infrequently attributed disadvantage to the socioeconomic circumstances which lead to increased risk of poor health. Instead, documents more commonly attributed disadvantage and vulnerability to individual factors such as lifestyle behaviours;

*“Within NSW there are sub-populations that warrant particular attention given their high prevalence of inadequate physical activity, unhealthy eating and higher than average rates of overweight and obesity” *(NSW Healthy Eating Active Living Strategy 2013-2018, p. 20).

 There were inconsistencies between policy documents in the proposal of actions for priority groups. This suggests that the rhetoric around priority groups was not always translated into policy action. Where documents did propose obesity prevention actions for priority populations, approaches were dominated by targeted education strategies. These representations reinforce the problematization of individual responsibility for overweight and obesity, suggesting that disparities within the population arise from a lack of knowledge and awareness. These representations also imply that the differences between population groups are due to the biology or behaviour of these groups rather than socially constructed as a result of unjust policies;

 “*There is a need for educational and incentive-based strategies to improve skills in buying and preparing healthy foods …By targeting better nutrition and physical health as part of its broader social equity agenda, the government will build on the wide range of programs already in place to assist those experiencing disadvantage”* (ACT Towards Zero Growth Healthy Weight Action Plan, p. 15).

###  6. Shared Responsibility for Childhood Obesity Prevention

 Childhood obesity was represented as the responsibility of multiple stakeholders. This was explicit in some documents which articulated a shared responsibility for health among individuals, society and governments, including a shared responsibility beyond the health sector;

*“Health is a shared responsibility between those who will benefit from making healthy choices (for example individuals, families and communities) and those who provide the infrastructure, services and support (governments at all levels, professional associations, the non-government sector, the research community, industry and business, and unions)” *(National Preventive Health Strategy: Australia the healthiest country by 2020, p. 41).

 “*…health is not merely a product of healthcare activities but is influenced by a wide range of social, economic, political, cultural and environmental factors, many outside the health sector” *(The Eat Well Be Active Strategy for South Australia 2011-2016, p. 53).

 One policy document from the ACT proposed to navigate the complexities of Australia’s federated system of government and identify opportunities for its government to regulate unhealthy food marketing.

 “*Australian experience suggests state or territory-based regulation of television advertising is problematic, however the ACT Government will examine its regulatory control across advertising mediums. There is a particular need to address marketing directed at children in close proximity to schools, playgrounds and child care centres*” (ACT Towards Zero Growth Healthy Weight Action Plan, p. 17).

 On the other hand, some states and territories shifted all responsibility to the federal government, as seen in the NT Nutrition and Physical Activity Strategy, deferring to national action on unhealthy food marketing;

 “*Contribute to national initiatives seeking to reduce exposure to advertising of energy dense, nutrient poor (EDNP) foods and drinks to children*” (Northern Territory Health Nutrition and Physical Activity Strategy 2015-2020, p. 33).

 The intent to work in partnership to address childhood obesity in Australia was evident throughout all policies, with partnership positioned as a guiding principle in a number of documents. Partnerships across levels of government, between government sectors and with external partners such as non-government organisations, academic institutions, industry and community were mentioned throughout policy documents, although roles and responsibilities of each partner were generally not articulated. The principle of working in partnership was supported by actions proposed to create and foster partnerships in order to achieve specific goals;

*“Strengthen partnerships with the sport and recreation sector to increase regular participation in sports and active recreation across the lifespan, and improve the supply and promotion of healthy food and drinks at sporting clubs” *(Queensland Healthy Weight Strategy 2017 to 2020, p. 8).

## Discussion

 This study presents the first critical analysis of how inequalities in childhood obesity are represented in Australian health policy. Our analysis examined the representations of inequalities in childhood obesity across a sample of 18 Australian health policy documents proposing action to prevent childhood overweight and obesity.

 Childhood overweight and obesity was predominantly problematized as an issue of individual responsibility. Actions proposed in policy documents focused primarily on information provision and education to improve knowledge and skills and change children and parents’ behaviour with a focus on increasing fruit and vegetable intake and physical activity, and decreasing unhealthy food consumption and sedentary behaviour. The framing of nutrition and obesity as issues of individual responsibility has been identified in other policy analyses^26,30,32^ and media studies.^33,44,45^ For example, Australian government healthy weight campaigns have frequently been built on the assumption that individuals are responsible for their own health and that behaviour change is a matter of individual choice.^45^ Governments have previously been observed to frame obesity as an issue of individual responsibility and to give preference to interventions targeted towards individuals.^23,46^ The framing of obesity as the responsibility of individuals aligns with a neoliberal ideology characterised by behavioural approaches with a minimal role for government intervention or industry regulation.^28^

 The way childhood obesity is framed in policy and by media has widespread impacts. Framing childhood obesity as an issue of individual responsibility is likely to garner less public support for government action to address obesity compared to societal responsibility frames.^47^ Such emphasis on individual responsibility by governments, without also addressing the underlying drivers of weight gain, is likely to exacerbate health inequalities^18^ as lifestyles are structurally determined, particularly among those with lower socioeconomic position.^35^ Actions that rely upon individual agency, such as behaviour change campaigns and promoting healthy eating guidelines are less likely to be effective among those with fewer social and economic resources, unless the structural barriers that constrain healthy eating such as access and affordability to healthy diets are addressed.^18^

 Policy documents acknowledged the social determinants of inequalities in childhood obesity in their rhetoric but not in their solutions, thereby problematizing childhood obesity as an issue of individual responsibility. The representations of individual responsibility for childhood obesity stand in contrast to the representations made by public health and academic community which represent obesity as an issue arising from social determinants.^26,33^

 In particular, we observed a focus on actions proposed in schools and other community settings. This settings-based problematization of childhood overweight and obesity could be interpreted to imply that settings are not doing enough to prevent childhood excess weight gain among children. On the other hand, it could be that governments are placing the onus on settings, such as schools to take responsibility for action to improve children’s’ health. Indeed, schools play in influential role in the development of children’s health behaviour and have been identified as highly capable of enacting policies to positively impact obesity.^48^ Community-based interventions are also increasingly being recognised for their role in improving childhood obesity outcomes.^49^ Whilst settings and community-based interventions have been shown to have positive outcomes across all socioeconomic groups,^50^ it is important that actions address the underlying determinants of inequalities in childhood overweight and obesity and are not limited to educational interventions that rely on individual agency for behaviour change.^18^

 Food access, food pricing and affordability, and unhealthy food marketing were acknowledged rhetorically in policy documents, but were not consistently committed to with proposed actions. These factors shape the environments in which food choices are made, and are arguably more influential than individual factors in the development of obesity.^11,18^ Where objectives to address the SDoH were proposed in policy documents, actions tended to focus on changing behaviour, rather than environments. This has been observed previously^51^ and has been described as ‘lifestyle drift,’ where policies acknowledge a broad range of determinants, yet actions focus on behavioural interventions.^52^ Similar representations of obesity have also been observed within Canadian government documents.^53^ A lack of focus on the SDoH in government policy silences the broader determinants of obesity and instead emphasises individual behaviour which may perpetuate weight stigma.^53^

 Furthermore, a lack of proposed action to address the underlying determinants of childhood obesity allows the individual responsibility frame to predominate throughout the policy documents. This has been observed elsewhere, with proposed solutions to obesity targeted towards individuals through health promotion and children’s education.^46^ In direct contrast, the public health sector frames the causes of and solutions to obesity in the context of the SDoH.^26^ This framing draws on evidence that indicates addressing underlying drivers of health is necessary to shape health-promoting environments which support, instead of undermine, healthy behaviours across populations.^9^ Creating environments that support fair opportunities for healthy behaviours for all will be necessary to achieving equitable reductions in childhood obesity.^18,35^ To do this requires government regulation.^17,48^ In this analysis, irrespective of the political party responsible for each policy document, we found very few documents that proposed regulatory action. This aligns with the findings of previous research that suggests regulatory action to address obesity has not been a political priority in Australia for a number of reasons including a neoliberal ideology that promotes individual responsibility (driven mainly by conservative governments), lack of political will to impose regulation (all governments), and food industry opposition (all governments).^20,43^

 Equity was positioned as a principle underpinning actions proposed within a majority of policy documents. However, references to equity were predominantly rhetorical. We observed limited reporting of inequalities in childhood obesity or inequalities in health more broadly. Instead, data were reported in aggregate, masking differences in health status within populations. This representation silences the disproportionate burden of obesity and associated ill health that is carried by groups experiencing greater socioeconomic disadvantage.^17^ Instead what is required is political commitment to ongoing monitoring and reporting of health status according to social group and action to address these disparities.^35^

 Limiting equity to an underlying principle of policy confines the discussion to a symbolic one.^35^ Without translation to more substantive policy commitments including clear actions and allocations of resources, this rhetoric is limited in its capacity to achieve equity.^28^ There is therefore a clear need to translate intentions around equity into action through higher levels of political commitment.^17,28^ Explicit actions to address health inequalities and their underlying determinants are considered essential to equity-oriented health policy^17,34,35^ however these were lacking in the policy documents examined in this study. In saying that, strategies to create healthy school food environments and policies that regulate unhealthy food marketing targeting children were identified in this analysis. These actions have the potential to change the environmental conditions in which children live, learn and play^51^ and are likely to have equitable impacts.^18^

 A number of priority populations were problematized throughout policy documents. Young children were represented as vulnerable to poor nutrition and the early childhood period was identified as a priority for action to address childhood obesity. Evidence demonstrates that conditions in early life are an important determinant of obesity,^9^ with early life a critical time for optimising healthy behaviours, through breastfeeding, the introduction of complementary foods, and the development of healthy eating, physical activity, and sleep patterns.^54^ The early childhood period is also critical in the development or avoidance of inequalities as the conditions to which children are exposed in early life will either promote or compromise health.^15^

 Aboriginal and Torres Strait Islander peoples, culturally and linguistically diverse communities and low-income families were also identified as priority populations in policy documents. These population groups were represented as experiencing overweight and obesity because of their poor lifestyle choices, rather than lacking privilege and adequate access to the resources required for good health.^55^ A similar representation was observed in Danish public health policy documents which represented poor health among populations experiencing disadvantage as caused by their own behaviour.^56^ This is in contrast to an equity-focused approach that identifies and names the conditions or circumstances that have led to individuals or groups of individuals being at greater risk of poor health.^57^ Representing vulnerable populations as a problem to be solved perpetuates a deficits-based perspective that may reinforce disadvantage.^58^ In the case of Aboriginal and Torres Strait Islander peoples’ health and wellbeing evidence shows that a self-determination and a strengths based approach is fundamental.^59^

 Objectives and actions to improve health among priority populations were articulated with a dominant focus on targeted approaches such as tailored education campaigns and support programs. This reinforces the notion of individual responsibility and represents inequalities in childhood obesity as a dichotomy, where a particular group is seen to be at greater risk compared to the rest of the population.^60^ A targeted approach such as this may reduce the inequality gap, but ignores the social gradient in health.^14^ An alternative to a targeted approach is a universal approach. This includes strategies that are aimed at an entire population, such as the promotion of dietary guidelines, or mandatory food and nutrition front-of-pack labelling. Although designed to reach the whole population, universal approaches may not necessarily have the same impact across population groups. For example, interventions that aim to provide information or education rely heavily on individual agency without addressing the underlying determinants of health inequalities.^18^ In between sits an approach known as proportionate universalism, where universal policies are designed and delivered at a scale and intensity relative to the level of need across populations.^36^ Ultimately, universal policies designed according to need, complemented by carefully designed targeted strategies, will be required to address childhood overweight and obesity across the entire socioeconomic gradient. Ongoing assessment of the impact of universal policies on socioeconomic inequalities will be necessary to ensure policies are having equitable impacts.^48^

 Policy documents emphasised that the health sector alone cannot resolve childhood obesity and partnerships were frequently mentioned. Yet the roles and responsibilities of other partners and the extent of their commitment were not described, and actions proposed within policy documents were confined to the health sector. This limits action to address the drivers of health outcomes that sit outside the health sector^36^ and could be perceived as a political tactic to delay action. Partnerships between sectors are necessary for two key reasons. First, responsibility for the determinants of inequalities in childhood overweight and obesity extend beyond the health sector and include housing, education, employment and access to health and social services.^9,22^ Second, food policy actions that address the upstream determinants of dietary behaviour require engagement from multi-sectoral partners. For example a health levy on sugar-sweetened beverages requires coordination between government health, finance, agriculture, trade and commerce ministries.^61^ In order to address inequalities in childhood obesity, health policies will need to actively engage stakeholders across sectors, and take stewardship of cross-sectoral actions that addresses the underlying social and commercial determinants.^35,62^

 There are some limitations to consider. Our study sought to analyse how inequalities in childhood obesity are represented in policy documents. Findings do not reflect the extent to which proposed actions have been implemented or evaluated. Our focus was on health policy documents as an indicator of governments’ rhetorical and institutional commitment to act on childhood obesity and its inequalities. The policy documents included in this study are the most recent of their type, however some have expired – such as Eat Well Australia: an agenda for action in public health nutrition 2000-2010. This illustrates an urgent need for comprehensive government action. Of note, a National Obesity Strategy is under development, tabled for release in 2021. Although all documents included in this study were published by government health departments, we recognise that strategies proposed by other government departments will also impact the determinants of childhood obesity and are likely to impact the widening or narrowing of health inequalities.^14^ It is possible that documents from other portfolios (eg, education) may have contained relevant actions but it is likely that these would have been cross-referenced in health policy documents. Analysis of representations of health equity in policy documents outside the health sector remains an important area for future research. Finally, it is important to note that the authors approached this analysis with a positionality stemmed in public health equity. In order to maintain rigour in our analysis, we used a theory-informed coding framework that was developed apriori and referred to the theory and evidence throughout the analytic process.

 This study also has a number of strengths. To our knowledge, this study is the first of its kind. Its findings improve understanding of how childhood obesity and related inequalities are represented in Australian health policy, and how these representations potentially limit the possible solutions for addressing inequalities in childhood obesity. The analysis illuminates silences in these representations such as a silencing of the existence and SDoH inequalities within and between population groups. Our findings suggest a role for advocacy and political leadership to challenge current representations and reframe the issue of obesity in a way that puts equity and the SDoH equity at the forefront of the policy agenda. This requires a paradigm shift from individual responsibility towards action to address the SDoH, to ensure that future policies are aptly designed to achieve the goal of reducing inequalities in childhood obesity.

## Conclusion

 Australian health policy documents predominantly represent childhood obesity as an issue of individual behaviour, with SDoH recognised but not sufficiently targeted with proposed actions. Equity is positioned as a rhetorical guiding principle, but this is not translated into actions that seek to achieve equity. These representations set an agenda for public health policy that is unlikely to address the underlying causes of inequalities in childhood obesity. In order to reduce inequalities in childhood obesity future health policies will require a greater focus on health equity and commitment to actions to address the SDoH. These findings highlight significant gaps in the current health policy landscape and can be used to inform the development of future policy and advocacy that aims to promote fairer opportunities for health across the population.

## Ethical issues

 As this research only involved analysis of publicly available, organisational documents institutional ethics approval was not required.

## Competing interests

 Authors declare that they have no competing interests.

## Authors’ contributions

 AC designed the study with input from all authors. AC, CZ, and JB developed the analytical framework. AC located and analyzed all policy documents with input from CZ and JB. All authors contributed to the interpretation of the results. AC prepared and revised the manuscript with contributions from all authors.

## Funding

 This work was supported by the Australian Government Research Training Program (AC & CZ) and the National Heart Foundation of Australia (Future Leader Fellowship 102047) (KB).
